# Computational Modeling and Treatment Identification in the Myelodysplastic Syndromes

**DOI:** 10.1007/s11899-017-0412-z

**Published:** 2017-09-14

**Authors:** Leylah M. Drusbosky, Christopher R. Cogle

**Affiliations:** Division of Hematology and Oncology, Department of Medicine, College of Medicine, University of Florida, 1600 SW Archer Road, Box 100278, Gainesville, FL 32610-0278 USA

**Keywords:** Myelodysplastic syndromes, Biomarkers, Computational biology, In silico

## Abstract

**Purpose of Review:**

This review discusses the need for computational modeling in myelodysplastic syndromes (MDS) and early test results.

**Recent Findings:**

As our evolving understanding of MDS reveals a molecularly complicated disease, the need for sophisticated computer analytics is required to keep track of the number and complex interplay among the molecular abnormalities. Computational modeling and digital drug simulations using whole exome sequencing data input have produced early results showing high accuracy in predicting treatment response to standard of care drugs. Furthermore, the computational MDS models serve as clinically relevant MDS cell lines for pre-clinical assays of investigational agents.

**Summary:**

MDS is an ideal disease for computational modeling and digital drug simulations. Current research is focused on establishing the prediction value of computational modeling. Future research will test the clinical advantage of computer-informed therapy in MDS.

## Introduction

Myelodysplastic syndromes (MDS) comprise a variety of clonal hematopoietic neoplasms with each showing dysplastic hematopoiesis, observed by light microscopy that cause at least one peripheral blood cytopenia and a risk for progression to acute myeloid leukemia (AML) [1–3]. MDS typically presents in older individuals and is the most common, age-associated clonal hematopoietic disease [4–6].

With advent of DNA sequencing, we now understand that most MDS cases harbor one or more of over 70 driver gene mutations in a subclonal architecture [7–9]. Furthermore, when using whole exome sequencing and array-based comparative genomic hybridization (CGH), we have detected unique constellations of hundreds of genomic abnormalities in our MDS patients, which brings to light the individual nature of MDS.

The necessity to understand MDS is impelled by a limited number of therapeutic options [10]. In the current era, where 50% of MDS patients transiently respond to hypomethylating agents, azacitidine or decitabine, and only 20% of non-del(5q) MDS respond to lenalidomide, there is an urgent need to predict which patients will achieve benefit from these agents, spare patients who will not, and identify alternative drugs. Furthermore, as MDS clones evolve after treatment interventions in individual patients, it is imperative for clinicians to see those MDS clonal dynamics so that appropriate revisions in treatment strategy can be made.

Thus, MDS is biologically complex and challenging to treat. Bridging MDS disease characteristics to clinical decision-making requires much greater sophistication than our wholesale treatment practices of the twentieth century.

## MDS as a Measurable Disease

Although MDS spent much of the twentieth century first as an *odo-leucoses* and then a refractory cytopenia distinguished from vitamin and mineral deficiencies [11], it was not until the very end of that century that clinicians began to measure its existence. In 1997, Peter Greenberg and collaborators synthesized quantifiable MDS disease characteristics from prior international efforts into a minimal set of measurable variables (i.e., number of cytopenias, bone marrow myeloblast percentage, and bone marrow chromosome karyotyping) that correlated with patient survival time and progression to AML [12]. The International Prognostic Scoring System (IPSS) was eventful on several levels, but chief among its significances was that it transformed MDS into a calculable disease. Revisions and adaptations of the IPSS have followed, primarily by adding clinical and laboratory variables found to correlate with survival based on multivariable logistic regression statistics or carving out select sub-populations of MDS patients [13, 14, 15•, 16, 17]. With the current application of genomics and other –omics technologies, the next waves of IPSS revisions will incorporate more molecular pathology.

Another key point here is that sharing of a robust MDS database was required to mature MDS management from a descriptive cytomorphologic disease to a measurable entity on which incremental improvements could be plied.

## MDS Biomarkers Correlating with Treatment Response

In addition to clinical factors, somatic gene mutations associate with overall survival in MDS patients, and can be integrated into prognostic models to augment their predictive value.

For example, in lower-risk MDS, *TP53* gene mutations were found in 13% of patients with a preponderance found in del(5q) patients (23.6%) compared to non-del(5q) patients (3.8%) [18]. Multivariate regression analysis identified *TP53* as an independent predictor for shortened progression-free survival and shortened overall survival in lower-risk MDS patients. Importantly, the variant allele frequency cutoff for that study was 6%. Extending these findings to treatment, in del(5q) MDS patients treated with lenalidomide, the presence of *TP53* mutations correlated with a lower response rate and a higher rate of progression to AML [19].

A study of 439 MDS patients interrogated for mutations in 111 cancer-relevant genes found that mutations in *EZH2*, *RUNX1*, *TP53*, *ETV6*, and *ASXL1* were independent predictors of poor survival in a multivariate analysis controlling for IPSS classification [20]. This large cohort of MDS patients further enabled the investigators to correlate genotype and phenotype characteristics, leading to associations between *RUNX1*, *TP53*, and *NRAS* mutations with severe thrombocytopenia and increased blast percentage, and the association of *TP53* mutations with complex karyotype.

A subsequent study performed targeted sequencing of 111 genes across 738 patients with MDS and identified recurrent driver mutations in 43 genes [7]. This study verified the poor prognostic impact of genes such as *RUNX1*, *ASXL1*, *TP53*, *SRSF2*, and *U2AF1* and also identified a correlation between the number of driver mutations present and leukemia-free survival. These clinical associations are excellent examples of how somatic gene mutation data build upon existing clinical risk-stratification scoring systems.

With regard to chemotherapy treatment, in patients whose MDS harbored a *TET2* mutation at > 10% variant allele frequency and wild-type *ASXL1*, they were 2.5 times more likely to achieve improved clinical outcomes after hypomethylating agent (HMA) treatment [21, 22, 23, 24•].

In terms of allogeneic hematopoietic cell transplantation (HCT), although transplant can bring about cure in approximately 40% of MDS patients, the countervailing risks of transplant-related death and MDS relapse after transplant necessitate careful patient selection. Many transplant centers base transplant eligibility on clinical and comorbidity factors, but recent evidence indicates that molecular biomarkers may also be useful in identifying MDS patients for allogeneic HCT. To identify these biomarkers, 288 MDS samples were retrospectively interrogated for the presence of mutations in 22 myeloid genes and then correlated with post-transplant outcomes [25]. Among the 22 genes, *EZH2*, *RUNX1*, *TP53*, and *ASXL1* were associated with worse transplant outcomes. A multivariable regression analysis incorporating clinical data further identified *EZH2* mutations as an independent poor prognostic factor.

In a separate retrospective MDS study, mutations in *TP53* and *TET2* were identified as biomarkers for poor clinical outcome after allogeneic HCT to such an extent that the strategy of allogeneic HCT in this patient population bears further study and optimization [26].

Another research effort sequenced 129 genes of 1514 MDS patient pre-transplant samples [27]. This study confirmed that *TP53* mutations, along with the p53 regulator *PPM1D* and *JAK2* mutations, were significantly associated with shortened overall survival time after transplant. Mutations in *TP53* and/or Ras pathway (*NRAS*, *KRAS*, *PTPN11*, *CBL*, *NF1*, *RIT1*, *FLT3*, and *KIT*) mutations were associated with shorter time to disease relapse after transplant.

Building upon with these molecular studies, others found that the presence of *TP53*, *RUNX1*, or *ASXL1* mutations impacted post-transplant survival when somatic gene mutations and IPSS-R risk scoring were combined [28]. This augmentation by molecular profiling supports the evolution of MDS clinical prognostic systems into a clinico-molecular system.

## The Upcoming Era of Computational Modeling for MDS

In these early days of correlative molecular studies driven primarily by multivariate regression statistics, the associations among genomic abnormalities and treatment responses are mounting in number and complexity. At the clinical practice level, it is challenging to remember and interpret the increasing number of significant biomarkers in addition to clinical factors. Revelation of these numerous complex associations is transforming MDS into its next era when computational methods will be necessary to understand each patient’s disease network and interactions with treatment options.

**Fig. 1:**
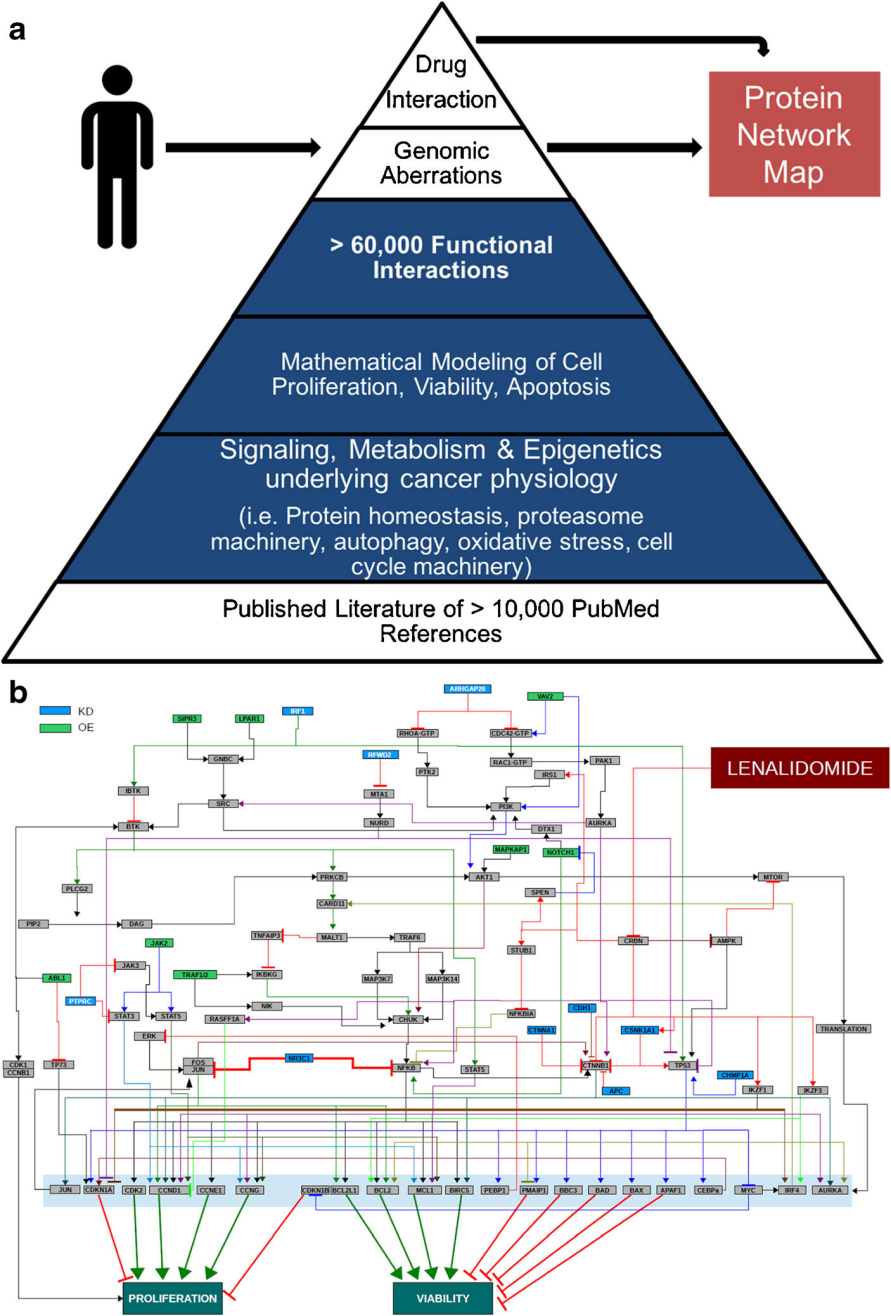
Multi-gene, multi-drug computational modeling in MDS. **a** The computational biology software was founded on PubMed references of intracellular elements involved in cancer cell physiology. Before inputting the MDS patient’s genomic abnormalities, the digital cell model was allowed to divide and die at a rate that was mathematically recorded over time and representative of a non-malignant state. Genomic abnormalities, such as gene mutations and gene copy number variations, from an MDS patient were then used to change the function of select protein networks. The rate of MDS cell division and death was then recalculated and compared to the non-malignant state. This change in MDS cell proliferation, viability, and apoptosis was expressed as a composite MDS cell growth score and represented the quantitative effect of the patient’s MDS mutanome. Drug and drug combinations were then modeled in the patient’s MDS network map to determine which drug or drug combination returns the MDS cell growth score back to rate of a non-malignant state. **b** This protein network map is from a patient with del(5q) MDS who did not achieve clinical improvement with lenalidomide. NGS and CNV data from the patient’s MDS cells were used to project a protein network map. Proteins are labeled as knock-down (KD, blue) or over-expressed (OE, green). Interacting proteins are depicted in gray. Downstream effects on MDS cell proliferation and viability are also mapped. A positive interaction is depicted with an arrow, whereas an inhibitor interaction is depicted as a bar. Lenalidomide (burgundy) is simulated as directly interacting with its target (CRBN). In this computational modeling and drug simulation, the patient’s MDS biology is predicted insensitive to lenalidomide because of watershed effects of increased beta-catenin activity and weakened TP53 activity

We recently tested a computational biology method that comprises software coding for 4700 intracellular pathway elements capable of simulating over 60,000 functional interactions, including coverage of the kinome, transcriptome, proteome, and metabolome (Cellworks Group, Inc.) (Fig. 1a) [29••]. The software coding was sourced from PubMed references over a 10-year period. The computer software first determines whether the patient’s MDS gene mutations result in activated or inactivated proteins, and then whether the protein is over-expressed or under-expressed by utilizing the patient’s MDS cytogenetics and/or chromosome copy number variation (CNV) data. Protein network maps of each patient’s MDS mutanome depict the interactive nature of all predicted aberrant protein signaling pathways (Fig. 1b).

Each patient’s map also enables a quantitative means of measuring the disease physiology in a composite MDS cell growth score consisting of MDS cell apoptosis, cell proliferation, and cell viability. Next, the patient’s MDS profile can be digitally screened to predict response to drugs of interest. If the drug targets and downstream mediators for the drug are present and unperturbed by genomic mutations in the MDS profile, the cell growth score will normalize in a dose-dependent manner, suggesting a response to that particular agent. If the drug in the model does not have its targets or necessary downstream mediators, then the MDS cell growth score is unchanged and the MDS is predicted as non-responsive to the drug. Combinations of drugs are also tested in any permutation. Once the model is created and validated over the course of 5–7 days, the drug simulations can be executed on the order of minutes. PubMed references are provided as documentation linking the genomic abnormalities with predicted drug sensitivity or resistance.

To test this computational method in MDS, we accessed three retrospective MDS datasets [29••]. The first cohort modeled 46 del(5q) MDS patients and predicted their response to lenalidomide treatment in a blinded fashion. When the simulated drug predictions were compared to clinical responses, 37/46 (80%) matched the observed clinical outcomes. Importantly, in del(5q) MDS cases that did not improve after lenalidomide treatment, the computational approach identified potential mechanisms for lenalidomide resistance (Fig. 1b). The second cohort in the study modeled 15 MDS patients treated with azacitidine and accurately predicted 12/15 (80%) clinical outcomes. The third cohort modeled 10 MDS patients treated with a combination of azacitidine and lenalidomide, and accurately predicted 10/10 (100%) of the clinical outcomes. Not only was this method accurate in predicting MDS patient responses to standard of care (SOC) therapy, it was able to highlight the drug sensitivity and resistance mechanisms for each case (Fig. 1b).

Based on these results, we subsequently initiated a prospective clinical trial designed to test the feasibility of computer-informed treatment in the management of MDS patients (ClinicalTrials.gov NCT02435550). The trial establishes the prediction values of the computational method and is generating IDE-enabling data for a future clinical trial randomizing HMA-refractory MDS patients to SOC versus computer-informed treatment. Preliminary data show high prediction accuracy in MDS and AML patients receiving SOC [30]. The PubMed references identified by the computational modeling and drug simulations are necessary for justifying medical necessity to health insurance carriers for drug coverage. This provision of PubMed references is a major difference between algorithmic methods and artificial intelligence systems that use heuristics, and thus demonstrates a real-world advantage to algorithmic methods.

Ultimately, this technology is meant to select MDS patients who will benefit most from treatment, spare patients from unwarranted toxicities in those who will not achieve benefit, and identify alternative treatments with greater chance for clinical improvement. These advantages are of interest to patients, clinicians, and payers such as health insurance carriers.

In addition to accurately discriminating responders from non-responders, this computational biology method has also been used to screen investigational drugs for application in MDS. With each MDS protein network map representing an MDS patient’s cell line, a database of 1000 MDS patients generates 1000 MDS cell lines with no laboratory-induced genomic artifacts. These digital MDS models can be organized into treat-naïve and HMA-refractory cohorts, thus enabling pre-clinical testing to specific MDS sub-populations for intended market approval. Because computational modeling does not require additional tissue or animal xenografting, a limitless number of drugs or drug combinations can be tested in the digital MDS models. Drugs such as BET inhibitors, CDK4/6 inhibitors, and venetoclax have been screened by this MDS computational modeling method with identification of certain genomic signatures that correlate with predicted efficacy [31, 32]. These drug-signature pairs inform eligibility criteria for precision enrollment clinical trials, companion diagnostics, and swift pathways to market approval.

## Data Need for Next Generation Computing in MDS

As –omics profiling becomes more affordable, clinical trials are increasingly incorporating these technologies. Thus, clinical trials provide a wealth of molecular and clinical data from cancer patients. The availability of publicly available, genomically annotated clinical databases derived from cancer clinical trials is perhaps just as important as the clinical trials’ primary objectives. The Cancer Genome Atlas (TCGA), Gene Expression Omnibus (GEO), cBioPortal, The Cancer Genomics Hub, canEvolve, and others have collected genomic information from a myriad cancer patients, although the lack of comprehensive clinical annotation and therapy response in most of these datasets is a major limitation [33].

Celgene’s Connect MDS/AML Disease Registry aims to fill this data gap by capturing patient demographic data, diagnostic laboratory and bone marrow pathology data, genomic mutations, prognostic risk variables, treatments, and clinical outcome data for 1500 newly diagnosed lower-risk MDS, higher-risk MDS, ICUS, and AML patients [34]. This rich dataset is expected to answer many current and future questions in MDS and AML.

Additionally, several groups have allowed access to their annotated clinical and/or genomic data, providing opportunities for novel computational analyses that will lead to important discoveries in MDS [7, 9, 35].

Ongoing clinical trials, such as those coordinated by the CIBMTR, are comparing outcomes with allogeneic HCT versus HMA therapy or best supportive care. Genomically annotated clinical records from these CIBMTR trials will be essential in assessing the potency of allogeneic HCT to overcome poor-risk MDS genetics.

Going forward, a concerted effort by an honest broker, such as a professional society, is needed to coalesce and harmonize the assortment of MDS datasets worldwide. This MDS database would engage engineers and scientists outside the traditional MDS research community and enable detection of a greater number MDS variables that associate with prognosis and treatment response.

## Conclusions

A major need in the treatment of MDS patients is to identify those with poor prognosis features who will benefit from therapy. The advent of NGS has made clear the importance of molecular profiling, as certain genomic aberrations significantly associate with drug response, survival, and allogeneic HCT outcomes. As the full complexity of MDS biology unveils, prognostic and predictive modeling will need to utilize sophisticated techniques aided by computational biology systems. By incorporating the totality of the MDS mutanome, computational methods have shown early accuracy in predicting drug response in patients with MDS. Use of a computational system may improve a clinician’s effectiveness in treating MDS, avoid toxicity when there is no prospect of benefit, weigh treatment options in the absence of guidelines, and find alternatives to treatment when none exist. Finally, computational modeling, unlike *ex vivo* assays and patient-derived xenograft (PDX) modeling, can rapidly test a limitless number of drugs and drug combinations, which catalyzes drug development for MDS.
